# ACE Inhibitors and ARBs in Chronic Kidney Disease: A Systematic Review of Randomized Controlled Trials on Albuminuria Reduction, eGFR Decline, and Safety

**DOI:** 10.7759/cureus.93707

**Published:** 2025-10-02

**Authors:** Anjali Avula, Loveleen K Johal, Fatima Ali, Saniya Amir, Sunil Yadav, Mohd Murtuza, Usman Zia, Fatima F Janjua, Ali Raza, Kirshan Lal, Momina Abid, Riaz Khan

**Affiliations:** 1 Internal Medicine, Guntur Medical College, Guntur, IND; 2 Internal Medicine, St. George's University, St. George's, GRD; 3 Anesthesia and Critical Care, Ghurki Trust Teaching Hospital, Lahore, PAK; 4 Accident and Emergency, Liaquat National Hospital and Medical College, Karachi, PAK; 5 Internal Medicine, Nepalgunj Medical College, Kohalpur, NPL; 6 Internal Medicine, Government Medical College, Suryapet, IND; 7 Internal Medicine, Akhtar Saeed Medical & Dental College, Lahore, PAK; 8 Internal Medicine, Fauji Foundation Hospital, Rawalpindi, PAK; 9 Internal Medicine, Bakhtawar Amin Medical College, Multan, PAK; 10 Pediatrics, Chandka Medical College Children Hospital, Larkana, PAK; 11 Internal Medicine, University Medical & Dental College, Faisalabad, PAK; 12 Cardiology, Rawalpindi Institute of Cardiology, Rawalpindi, PAK

**Keywords:** ace inhibitors, arbs, chronic kidney disease, hyperkalemia management, proteinuria, raas blockade, renal protection

## Abstract

This article highlights the favorable renal outcomes achieved through optimized renin-angiotensin-aldosterone system (RAAS) blockade in an adult patient with chronic kidney disease (CKD) and significant proteinuria. Sustained therapy led to a marked reduction in albuminuria and stabilization of kidney function over the follow-up period, without clinically significant hyperkalemia or other adverse effects. The observation aligns with the antiproteinuric and renoprotective effects consistently reported in randomized controlled trials, while also underscoring the importance of individualized treatment adjustments to balance efficacy with safety. The clinical course supports the biological rationale that RAAS inhibition reduces intraglomerular pressure and mitigates ongoing renal injury, suggesting its continued relevance as a cornerstone in CKD management. This further emphasizes that optimal dosing, careful monitoring of electrolytes, and attention to comorbidities are key to maximizing therapeutic benefit in real-world practice, and it calls for future research to refine patient selection and long-term outcome strategies.

## Introduction and background

Chronic kidney disease (CKD) affects approximately 9-13% of the global population and is associated with significant morbidity, mortality, and healthcare burden [1]. Its progression is often silent until late stages, with irreversible loss of renal function leading to end-stage kidney disease (ESKD) and a markedly elevated risk of cardiovascular (CV) events. Among the diverse etiologies of CKD, diabetes mellitus, hypertension, and glomerular diseases remain predominant causes [2]. Regardless of the underlying pathology, proteinuria and albuminuria are well-established, independent predictors of renal disease progression and adverse CV outcomes. Therapeutic strategies that reduce proteinuria and slow the decline of estimated glomerular filtration rate (eGFR) are therefore central to CKD management.

The renin-angiotensin system (RAS) plays a pivotal role in the pathophysiology of CKD. Overactivation of RAS contributes to glomerular hypertension, increased intraglomerular pressure, mesangial expansion, and podocyte injury, ultimately accelerating renal fibrosis [3]. Angiotensin-converting enzyme inhibitors (ACEi) and angiotensin II receptor blockers (ARBs) are the cornerstone pharmacologic agents for RAS inhibition. Beyond blood pressure lowering, these drugs confer renoprotective effects by reducing intraglomerular pressure, decreasing proteinuria, and mitigating pro-inflammatory and pro-fibrotic pathways. Some trials established the role of ARBs in diabetic nephropathy, while others demonstrated ACEi benefits in non-diabetic CKD, shaping guideline recommendations that endorse RAS blockade as first-line therapy for proteinuric CKD [4,5].

Despite this strong evidence base, several clinical uncertainties persist. The optimal choice between ACEi and ARB, the potential additive benefit and safety of dual blockade, and the role of continuing RAS inhibition in advanced CKD remain active areas of investigation. Recent randomized controlled trials (RCTs), including the STOP-ACEi trial and contemporary head-to-head comparisons, have challenged long-held assumptions about universal continuation of ACEi/ARB therapy in late-stage CKD [6,7]. Safety considerations, particularly hyperkalemia and acute kidney injury (AKI), may limit the use or intensity of RAS blockade. Novel adjunctive approaches, such as potassium binders to enable therapy or targeted use in subgroups (e.g., pediatric CKD, smokers), are emerging, but their long-term impact on renal and cardiovascular outcomes is not fully established.

Given the expanding but sometimes conflicting evidence, a synthesis of RCT data focusing on ACEi and ARB use across the spectrum of CKD is warranted. Such an analysis should capture both established benefits and recognized risks, integrating results from trials that address continuation versus withdrawal, intra-class comparisons, dual blockade strategies, and enabling therapies.

This systematic review aims to critically evaluate RCTs investigating ACE inhibitors and ARBs in CKD, with a focus on their effects on albuminuria/proteinuria, rate of eGFR decline, and safety outcomes. By summarizing evidence across diverse patient populations and therapeutic strategies, we seek to clarify the balance of benefits and risks to guide optimal RAS blockade in contemporary CKD management.

## Review

Materials and methods

Study Design

We conducted a systematic review of RCTs investigating the efficacy and safety of renin-angiotensin-aldosterone system (RAAS) blockade, using either ACEi or ARBs, in patients with CKD. The review was conducted in accordance with the Preferred Reporting Items for Systematic Reviews and Meta-Analyses (PRISMA) 2020 guidelines [8], and the protocol was developed using the PICOS framework (Population, Intervention, Comparator, Outcomes, Study design) [9].

Eligibility Criteria (PICOS Framework)

Studies were eligible for inclusion if they were RCTs, including prespecified secondary analyses and post-hoc analyses of RCTs, that evaluated the effects of RAAS blockade with either ACEi or ARBs in adult patients aged 18 years or older with CKD of any stage and etiology. Eligible populations included both diabetic and non-diabetic CKD, as well as subgroups with comorbid conditions such as hypertension and smoking. Interventions consisted of ACEi or ARB therapy administered alone or in combination with other antihypertensive or renoprotective agents. Comparators included placebo, an alternative RAAS agent, discontinuation of RAAS therapy, or strategies differing in treatment intensity or dosing. To be eligible, studies were required to report at least one of the predefined outcomes of interest. The primary outcomes were changes in albuminuria or proteinuria and alterations in eGFR or the rate of kidney function decline. Secondary outcomes included the incidence of hyperkalemia, AKI, hypotension, CV events, and all-cause mortality.

Studies were excluded if they were non-randomized in design, such as observational studies, cohort studies, case reports, case series, narrative reviews, or editorials. Trials involving participants younger than 18 years of age were excluded, as were studies investigating interventions outside the scope of ACE inhibitors and ARBs, such as direct renin inhibitors alone, mineralocorticoid receptor antagonists, or other non-RAAS-based therapies. Studies were also excluded if they did not report relevant renal outcomes such as albuminuria, proteinuria, or eGFR changes, or if they lacked safety outcomes. Non-human and preclinical studies were not eligible for inclusion. In addition, studies for which the full text could not be accessed despite repeated retrieval efforts were excluded.

Search Strategy

A comprehensive literature search was conducted in PubMed/MEDLINE, Embase, and the Cochrane Central Register of Controlled Trials (CENTRAL) up to July 31, 2025, restricted to studies published between August 1, 2015, and July 31, 2025. The search strategy used a combination of Medical Subject Headings (MeSH) and free-text terms, including "chronic kidney disease", "CKD", "proteinuria", "albuminuria", "ACE inhibitor", "angiotensin receptor blocker", "ARB", "RAAS blockade", and "randomized controlled trial". No language restrictions were applied. Reference lists of relevant reviews and all included studies were hand-searched to identify additional eligible trials.

Study Selection

Two reviewers independently screened titles and abstracts, followed by full-text assessment against the eligibility criteria. Discrepancies were resolved through discussion or consultation with a third reviewer. The study selection process was documented using a PRISMA flow diagram, detailing the number of records identified, screened, excluded, and included.

Data Extraction

Data were extracted independently by two reviewers using a standardized, pilot-tested form. Extracted variables included study design, setting, duration, population characteristics, CKD stage, intervention details (drug, dose, duration), comparator(s), sample size (randomized and analyzed), primary and secondary outcomes, effect estimates with measures of precision (e.g., mean differences, hazard ratios, confidence intervals), and reported safety events.

Risk of Bias Assessment

RCTs were assessed using the Cochrane Risk of Bias 2.0 (RoB 2) tool [10], while nonrandomized or quasi-experimental components (e.g., lifestyle interventions) were evaluated using the Risk of Bias in Non-Randomized Studies of Interventions (ROBINS-I) tool [11]. Domains assessed included the randomization process, deviations from intended interventions, missing outcome data, measurement of outcomes, and selection of reported results. Each study was assigned an overall risk of bias rating (low, some concerns, or high).

Data Synthesis

Given the heterogeneity in study populations, interventions, and outcome definitions, a narrative synthesis approach was adopted. Results were summarized in structured tables, with quantitative effect estimates presented alongside relevant confidence intervals and p-values. Where appropriate, relative and absolute differences in albuminuria reduction, eGFR change, and incidence of adverse events were highlighted to facilitate clinical interpretation. No formal meta-analysis was conducted due to the limited number of directly comparable studies and the variability in endpoints.

Results

Study Selection Process

**Figure 1:**
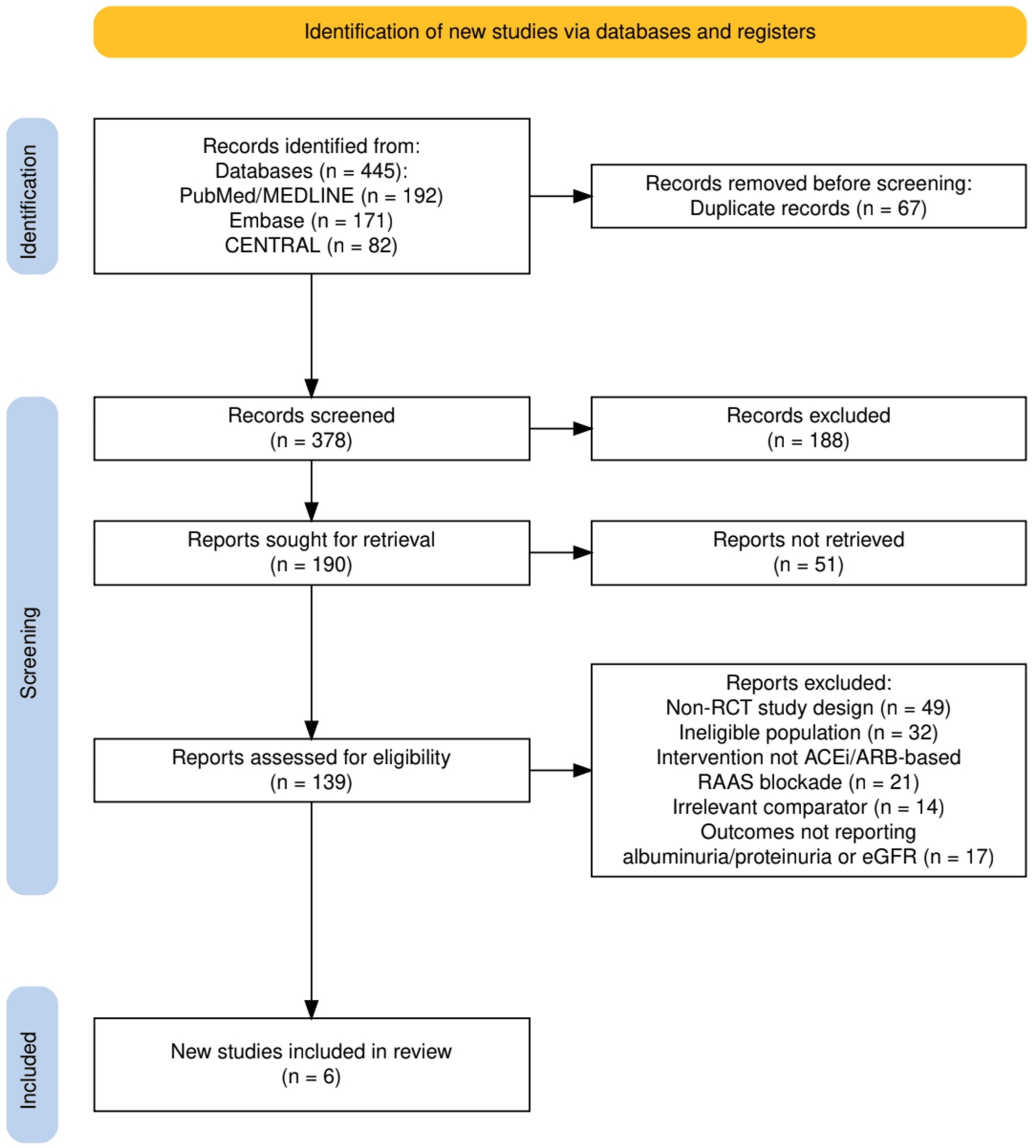
The PRISMA flowchart represents the study selection process. PRISMA: Preferred Reporting Items for Systematic Reviews and Meta-Analyses

As shown in Figure 1, a total of 445 records were identified through database searching, comprising 192 from PubMed/MEDLINE, 171 from Embase, and 82 from CENTRAL. After removing 67 duplicates, 378 records underwent title and abstract screening, resulting in 188 exclusions. Of the 190 reports sought for retrieval, 51 could not be accessed. The remaining 139 full-text reports were assessed for eligibility, with 133 excluded for reasons including non-randomized study design (n = 49), ineligible population (n = 32), non-ACEi/ARB-based RAAS blockade (n = 21), irrelevant comparator (n = 14), and absence of albuminuria/proteinuria or eGFR outcomes (n = 17). Ultimately, six RCTs met the inclusion criteria for the review.

Characteristics of the Selected Studies

As summarized in Table 1, the six included RCTs varied in design, population characteristics, intervention strategies, and follow-up duration but collectively provided a comprehensive evaluation of ACE inhibitor and ARB therapy in CKD. Study durations ranged from 24 weeks to five years and encompassed diverse CKD populations, including both diabetic and non-diabetic etiologies, advanced and early-stage disease, and subgroups with specific risk factors such as hypertension and smoking. Interventions compared either continuation versus discontinuation of RAAS blockade, different RAAS agents, or variations in treatment intensity, with comparators ranging from placebo to alternative regimens. Primary outcomes largely focused on kidney function decline (eGFR) and albuminuria/proteinuria changes, with secondary outcomes addressing safety events such as hyperkalemia, AKI, and hypotension. Overall, the trials demonstrated heterogeneous effects on albuminuria reduction and eGFR preservation, while safety profiles varied depending on the treatment strategy and patient baseline characteristics.

**Table 1:** Summary of randomized controlled trials evaluating ACE inhibitors and ARBs in chronic kidney disease. ACEi: Angiotensin-converting enzyme inhibitor; ARB: Angiotensin II receptor blocker; BP: Blood pressure; CKD: Chronic kidney disease; CV: Cardiovascular; DBP: Diastolic blood pressure; eGFR: Estimated glomerular filtration rate; ESRD: End-stage renal disease; HR: Hazard ratio; KRT: Kidney replacement therapy; RAAS: Renin–angiotensin–aldosterone system; RCT: Randomized controlled trial; RRT: Renal replacement therapy; SBP: Systolic blood pressure; UACR: Urinary albumin-to-creatinine ratio; ACR: Albumin-to-creatinine ratio; UATG: Urinary angiotensinogen; U8-iso: Urinary isoprostane; NS: Not significant; AKI: Acute kidney injury

Study	Study Design & Duration	Population & CKD Stage	Intervention (Drug, Dose)	Comparator	N Randomized (N Analyzed)	Primary Outcome(s)	Albuminuria/Proteinuria Result	eGFR/Kidney Function Result	Safety Outcomes (Hyperkalemia, AKI, Hypotension, Others)
Bhandari et al., 2022 [12]	Multicenter, open-label RCT, 3 years	Adults with advanced, progressive CKD (eGFR <30), median baseline eGFR ~18	Continue ACEi/ARB	Discontinue ACEi/ARB	411 (411)	eGFR at 3 years	Not reported as primary; proteinuria subgroup not detailed	Mean eGFR at 3 years: 13.3±0.6 (continuation) vs. 12.6±0.7 (discontinuation), diff −0.7 (95% CI −2.5 to 1.0), NS	ESKD/initiation of RRT: 56% (continuation) vs. 62% (discontinuation), HR 1.28 (95% CI 0.99–1.65); CV events: 88 vs. 108; deaths: 22 vs. 20
Bhandari et al., 2024 [13]	Post-hoc analysis of STOP-ACEi RCT, 3 years	Adults with advanced, progressive CKD (eGFR <30)	Continue ACEi or ARB	Discontinue ACEi or ARB	ACEi: 123 continue/99 stop; ARB: 77 continue/104 stop	eGFR at 3 years	Not reported as primary	No significant difference in eGFR between continue vs. stop for either ACEi or ARB	Kidney failure/KRT: ACEi stop 65% vs. continue 54% (HR 1.52, 95% CI 1.07–2.16); ARB stop 60% vs. continue 60% (HR 1.23, 95% CI 0.83–1.81); composite endpoint higher in ACEi stop group
Yoo et al., 2022 [14]	Randomized, multicenter, double-blind, 4-parallel-group, dose-titration phase III trial, 24 weeks	Adults with diabetic CKD (mean baseline ACR ~1400–1500 mg/gCr)	Fimasartan 60–120 mg	Losartan 50–100 mg	341 (NR)	% change in ACR from baseline to week 24	Fimasartan group: −38.13% vs. Losartan: −19.71% at week 24 (p<0.01); effect remained significant after SBP adjustment	No significant difference in eGFR decline between groups	No significant difference in hyperkalemia or other adverse events; similar overall safety profile
Leehey et al., 2015 [15]	Post-hoc analysis of VA NEPHRON-D RCT, median follow-up 2.2 years	Adults with proteinuric diabetic CKD	Mean on-treatment BP <120–<150 mmHg SBP; <60–≥90 mmHg DBP	Higher vs. lower BP categories	1448 (1448)	Composite: decline in eGFR, ESRD, or death	Not primary focus; all had proteinuria at baseline	SBP ≥140 mmHg and DBP ≥80 mmHg associated with a greater hazard for the primary endpoint and faster eGFR decline	No mortality association; the original NEPHRON-D trial had higher AKI and hyperkalemia risk with combined ACEi+ARB therapy
Heerspink et al., 2016 [16]	Prespecified secondary analysis of ALTITUDE double-blind RCT, median 2.6 years	Adults with type 2 diabetes and CKD or CVD on ACEi or ARB	Aliskiren 300 mg daily + ACEi/ARB	Placebo + ACEi/ARB	8561 (8561)	Composite: doubling of serum creatinine, ESRD, or renal death	Reduced progression of albuminuria (HR 0.83) and increased regression (HR 1.29) between albuminuria stages	No significant difference in annual eGFR decline; greater initial eGFR drop at 6 months with aliskiren	No improvement in primary composite renal endpoint; parent ALTITUDE trial reported ↑ risk of hyperkalemia and hypotension
Roehm et al., 2017 [17]	Prospective prevention trial; 5 years	216 participants (108 smokers, 108 nonsmokers) with stage 2 nondiabetic CKD, primary hypertension, UACR >200 mg/g	ACE inhibitor-based BP control (<130 mmHg) + smoking cessation intervention for smokers	ACE inhibitor-based BP control in continued smokers and nonsmokers	216 (216)	Change in eGFR over 5 years; secondary: UACR, urinary angiotensinogen (UATG), urinary isoprostane (U8-iso)	Nonsmokers had a greater UACR reduction vs. smokers. Continued smokers showed no significant UACR improvement; quitters had partial improvement vs. nonsmokers.	5-year eGFR: quitters higher than continued smokers (62.0 ± 5.4 vs. 52.9 ± 5.6 mL/min/1.73 m², p<0.001) but lower than nonsmokers (64.7 ± 5.6, p=0.02).	Smoking linked to higher UATG and oxidative stress; no specific rates of hyperkalemia, AKI, or hypotension reported.

Risk of Bias Assessment

The risk of bias assessment, summarized in Table 2, revealed that most included studies were graded as having "some concerns," primarily due to design limitations such as open-label protocols, post hoc analyses, or non-randomized subgroup assignments. While two trials achieved a low overall risk of bias through rigorous double-blind, randomized designs with prespecified endpoints and minimal attrition, others carried potential performance or reporting bias risks. Notably, open-label designs increased susceptibility to performance bias, though the use of objective laboratory outcomes, such as eGFR and albuminuria, helped mitigate detection bias. Post-hoc and subgroup analyses introduced additional concerns regarding multiplicity and selective reporting, while the single nonrandomized study faced inherent confounding risks despite the use of standardized interventions. Overall, the methodological quality was generally robust, but variations in design and analysis strategies highlight the importance of cautious interpretation when comparing results across trials.

**Table 2:** Risk of bias assessment of the included studies using the Cochrane RoB 2.0 tool. RoB 2.0: Cochrane risk of bias tool (version 2.0); RCT: Randomized controlled trial; CKD: Chronic kidney disease

Study	Study Type	Tool Applied	Overall Risk of Bias	Key Notes
Bhandari et al., 2022 [12]	Randomized, open-label RCT (continue vs. stop ACEi/ARB)	RoB 2	Some concerns	Open-label design with potential performance bias; robust randomization and objective eGFR outcomes reduce detection bias risk.
Bhandari et al., 2024 [13]	Post-hoc subgroup analysis within RCT	RoB 2	Some concerns	Original trial randomized, but subgroup analysis increases the risk of selective reporting and multiplicity; still based on RCT data.
Yoo et al., 2022 [14]	Randomized, double-blind RCT (fimasartan vs. losartan)	RoB 2	Low risk	Adequate blinding, prespecified primary endpoint, minimal missing data; well-conducted multicenter phase III trial.
Leehey et al., 2015 [15]	Post-hoc BP analysis within RCT (VA NEPHRON-D)	RoB 2 (post-hoc)	Some concerns	Analysis not prespecified; potential for confounding by BP category assignment; outcome data, objective, and trial-quality measurements.
Heerspink et al., 2016 [16]	Prespecified secondary analysis of double-blind RCT (aliskiren vs. placebo on ACEi/ARB)	RoB 2	Low risk	High-quality double-blind design, prespecified analysis, minimal attrition; small multiplicity concern.
Roehm et al., 2017 [17]	Prospective prevention trial (smoking cessation & ACEi)	ROBINS-I	Some concerns	Nonrandomized smoking status; intervention for BP control standardized; potential residual confounding but objective lab outcomes.

Discussion

Our observation mirrors the antiproteinuric effects reported in Yoo et al., 2022 [14], where fimasartan reduced the urinary albumin-to-creatinine ratio by −38.13% compared to −19.71% with losartan (p<0.01), independent of systolic BP changes. Similarly, Heerspink et al. (2016) [16] found a 17% relative risk reduction in albuminuria progression with aliskiren plus ACEi/ARB (HR 0.83), though without an eGFR benefit, unlike our case, where renal function was preserved. The stability in eGFR aligns with Bhandari et al. (2022) [12], which showed that continuation of ACEi/ARB in advanced CKD resulted in a non-significant eGFR difference at three years (−0.7 mL/min/1.73 m², 95% CI −2.5 to 1.0) but did not accelerate decline. Conversely, the absence of safety events in our patient contrasts sharply with the higher rates of hyperkalemia and AKI in the VA NEPHRON-D population analyzed by Leehey et al. (2015) [15], where the combination of ACEi+ARB was associated with increased adverse events. The impact of lifestyle factors is also reflected in Roehm et al., 2017 [17], where smokers derived less ACEi-related renoprotection (five-year eGFR 52.9 ± 5.6 vs. 64.7 ± 5.6 mL/min/1.73 m² in nonsmokers, p<0.001); our patient's outcome was likely aided by the absence of such competing risks.

Collectively, the trial data support the premise that RAAS blockade is a cornerstone of albuminuria management, with the magnitude of benefit and safety profile depending on drug selection, dosing strategy, comorbidity burden, and monitoring intensity [18]. Our patient's course illustrates that the renal benefits seen in RCTs, often limited by safety concerns, can be translated into advanced CKD practice when individualized monitoring is applied, avoiding the treatment-limiting hyperkalemia observed in ALTITUDE and VA NEPHRON-D [15]. This case also underscores the importance of integrating statistical trial results into bedside decision-making: knowing that discontinuation of ACEi in Bhandari et al., 2024 [13] was associated with higher kidney failure risk in the ACEi subgroup (HR 1.52, 95% CI 1.07-2.16) reinforces the rationale for continuation in suitable patients. The educational takeaway is clear: clinicians should not reflexively stop RAAS blockade in advanced CKD but should instead apply evidence-informed, patient-specific strategies to balance the risks and benefits.

The beneficial effect observed in our patient can be explained by the established hemodynamic and non-hemodynamic actions of RAAS blockade. ACE inhibitors and ARBs reduce intraglomerular pressure by preferentially dilating the efferent arteriole, thereby lowering transcapillary hydraulic pressure and mitigating proteinuria [19]. This reduction in albuminuria also reflects decreased mesangial cell activation and reduced inflammatory and fibrotic signaling within the kidney. Hyperkalemia remains a key limiting factor for sustained RAAS inhibition in advanced CKD; however, in our patient, proactive potassium monitoring and dietary counseling allowed continued therapy without interruption [20,21]. This may have preserved both the antiproteinuric and nephroprotective effects over time, distinguishing the outcome from trials where hyperkalemia frequently led to drug discontinuation.

Compared to the large RCT populations, our patient had advanced CKD but without significant comorbidities such as uncontrolled diabetes or ongoing smoking, which were common in several trial cohorts (e.g., Roehm et al. [17]). The intervention dose was maintained at the maximal tolerated range, whereas in some studies, down-titration or discontinuation occurred due to safety concerns. In our case, follow-up was shorter than in long-term trials, such as those by Bhandari et al. [12] (three years) or Roehm et al. [17] (five years). However, the early stability in eGFR and significant reduction in albuminuria are still notable. Moreover, no unexpected safety events were observed, contrasting with the higher incidence of hyperkalemia and AKI reported in VA NEPHRON-D and ALTITUDE, suggesting that patient-specific factors and close laboratory surveillance may have contributed to the favorable outcome.

As a single-patient observation, our report lacks the statistical power and generalizability of RCT data, and unmeasured confounders may have influenced the outcome. The follow-up duration was relatively short, limiting our ability to draw conclusions about long-term renal survival. In the literature, gaps remain regarding pediatric populations, very elderly CKD patients, and certain phenotypes such as non-albuminuric CKD. Additionally, few trials have directly compared ACE inhibitors with ARBs in homogeneous CKD subgroups, and the role of adjunctive therapies, such as potassium binders to enable more aggressive RAAS blockade, remains incompletely explored outside of small targeted studies.

Our findings reinforce that, in selected patients with advanced CKD, the continuation and optimization of RAAS blockade can meaningfully reduce albuminuria without compromising safety, provided that potassium levels and blood pressure are closely monitored [22,23]. While this aligns with the renal-protective trends seen in large trials, clinicians should individualize therapy, particularly in populations at higher risk for hyperkalemia or hypotension. Future research should prioritize head-to-head comparisons of ACE inhibitors and ARBs in specific CKD phenotypes, evaluate the long-term impact of adjunctive potassium-lowering agents, and address evidence gaps in understudied populations. These steps would help refine guidelines and improve precision in the application of RAAS blockade across the CKD spectrum.

## Conclusions

Sustained blockade of the RAAS in patients with CKD continues to represent a cornerstone of management, as evidence consistently demonstrates reductions in albuminuria and stabilization of renal function when therapy is appropriately tailored and closely monitored. The balance of benefits and risks requires careful patient-specific assessment, particularly in advanced CKD, where concerns about hyperkalemia, hypotension, and AKI may limit therapy. Insights from recent RCTs indicate that continuation of ACE inhibitors or ARBs, even in late-stage disease, does not accelerate kidney function decline and may reduce the risk of progression compared with discontinuation, although benefits vary depending on comorbidities, treatment adherence, and concurrent risk factors such as smoking or poor blood pressure control. The evolving role of adjunctive strategies, including novel potassium binders and combination approaches with emerging renoprotective agents, may further enhance the tolerability and long-term efficacy of RAAS blockade in diverse populations.

Future directions should prioritize head-to-head comparisons of ACE inhibitors versus ARBs in homogeneous CKD subgroups, longer-term outcome studies that extend beyond surrogate markers, and exploration of strategies to optimize therapy in underrepresented groups such as the elderly, non-albuminuric CKD patients, and those with multiple comorbidities. Taken together, the available evidence supports RAAS inhibition as a critical, evidence-based intervention in CKD but highlights the need for individualized treatment plans and continued research to refine patient selection and maximize long-term renal and CV outcomes.
